# Supplementary material for: Trends, geographical variation and factors associated with prescribing of gluten-free foods in English primary care: a cross-sectional study
Source: BMJ Open. 2018 Apr 16;8(3):e021312. doi: 10.1136/bmjopen-2017-021312 (PMC5905743; doi:10.1136/bmjopen-2017-021312)
Supplement: Supplementary data [file bmjopen-2017-021312supp001.pdf]

Appendix A - Gluten free prescribing BNF code list

0904010AK G/F /W/F /L/P Cooking Aids
0904010I0 Gluten Free Biscuits
0904010H0 Gluten Free Bread
0904010L0 Gluten Free Cakes/Pastries
0904010J0 Gluten Free Grains/Flours
0904010Z0 Gluten Free/Low Protein Biscuits
0904010F0 Gluten Free/Low Protein Bread
0904010AE Gluten Free/Low Protein Cereals
090401050 Gluten Free/Low Protein Cooking Aids
0904010V0 Gluten Free/Low Protein Grains/Flours
090401060 Gluten Free/Low Protein Meals
0904010E0 Gluten Free/Low Protein Mixes
0904010T0 Gluten Free/Low Protein Pasta
0904010AF Gluten Free/Low Protein Sweet/Savoury
090401030 Gluten Free Mixes
0904010Q0 Gluten Free Pasta
0904010A0 Gluten Free/Wheat Free Biscuits
0904010U0 Gluten Free/Wheat Free Bread
0904010AD Gluten Free/Wheat Free Cakes/Pastries
0904010AC Gluten Free/Wheat Free Cereals
0904010AB Gluten Free/Wheat Free Cooking Aids
090401070 Gluten Free/Wheat Free Grains/Flours
0904010AI Gluten Free/Wheat Free/Low Protein Bisc
0904010AJ Gluten Free/Wheat Free/Low Protein Mixes
0904010AH Gluten Free/Wheat Free/Low Protein Pasta
0904010AG Gluten Free/Wheat Free Meals
0904010AA Gluten Free/Wheat Free Mixes
090401080 Gluten Free/Wheat Free Pasta
0904010AU Gluten Free/Wheat Free Snacks
