# Supplementary material for: Trends, geographical variation and factors associated with prescribing of gluten-free foods in English primary care: a cross-sectional study
Source: BMJ Open. 2018 Apr 16;8(3):e021312. doi: 10.1136/bmjopen-2017-021312 (PMC5905743; doi:10.1136/bmjopen-2017-021312)
Supplement: Supplementary data [file bmjopen-2017-021312supp002.pdf]

Appendix B: level of missing data within dataset

Variable	Missing values	% missing
Composite measure score	29	0.4%
QOF score	0	0.0%
IMD score	1	0.0%
Practice list size	0	0.0%
Dispensing practice status	0	0.0%
% of patients over 65 years	1	0.0%
% of patients with long term health conditions	6	0.1%
CCG identifier	0	0.0%
**Practices with missing data for any variable**	35	0.5%
